# Accelerated phase contrast imaging using compressed sensing with complex difference sparsity

**DOI:** 10.1186/1532-429X-14-S1-W24

**Published:** 2012-02-01

**Authors:** Yongjun Kwak, Seunghoon Nam, Kraig V Kissinger, Beth Goddu, Lois A Goepfert, Warren J Manning, Vahid Tarokh, Reza Nezafat

**Affiliations:** 1 Department of Medicine (Cardiovascular Division), Beth Israel Deaconess Medical Center, Boston, MA, USA; 2 Engineering Sciences, Harvard School of Engineering and Applied Sciences, Cambridge, MA, USA; 3 Radiology, Beth Israel Deaconess Medical Center, Boston, MA, USA

## Background

One of the major limitations of phase contrast imaging is its long scan time. Compressed sensing (CS) has been recently utilized to reduce the scan time in phase contrast MR [1-3]. In this study, we propose an accelerated phase contrast MR approach in which sparsity of the complex difference (CD) image is used as the sparsifying transform to improve image reconstruction.

## Methods

**Figure 1:**
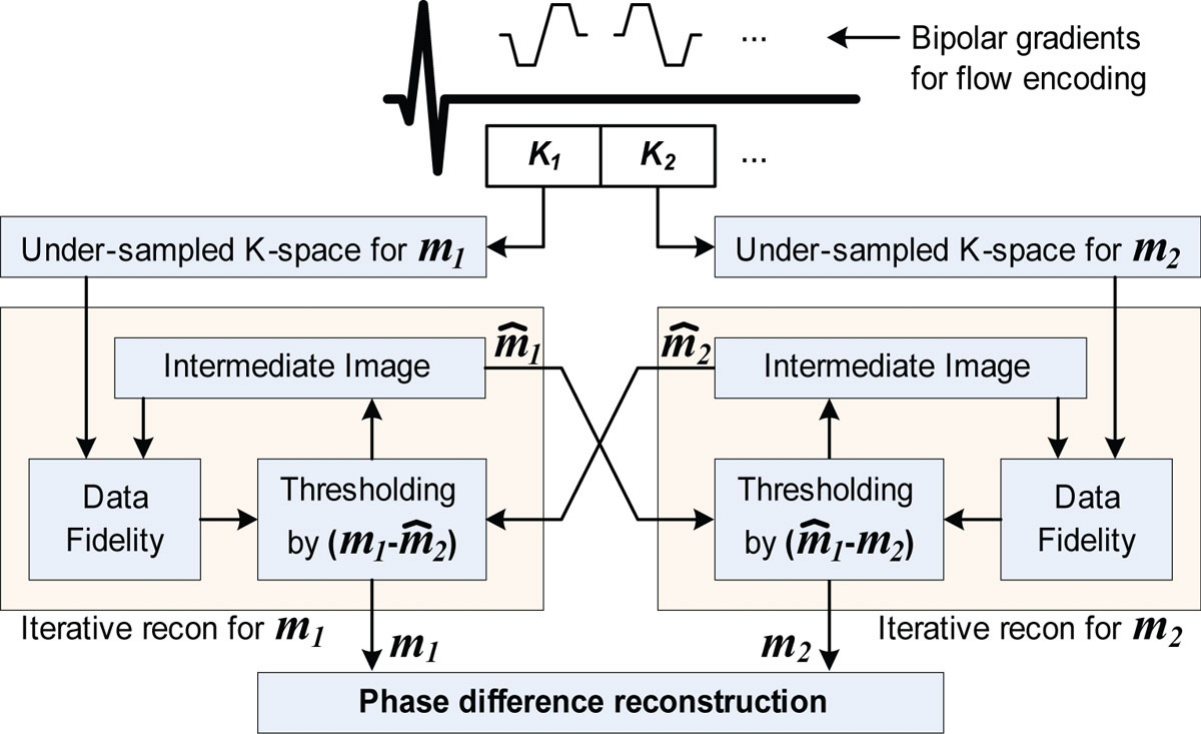
The proposed CS reconstruction algorithm for phase contrast MRI using complex-difference sparsity.

Figure 1 shows the proposed CS reconstruction with CD sparsity for phase contrast MR. The objective function *J* = *‖F_Ω_m_i_-y_i_‖_2_*+*λ‖Ψm_i_‖_1_‖*+*λ_CD_‖m_1_-m_2_‖_1_*, where *m_i_*_=_*_1_*_,_*_2_* are the two bipolar encoding acquisitions and *|m_1_-m_2_|* is the corresponding CD image, is used in the reconstruction. The reconstruction performs iteratively between two bipolar images. Every iteration, the recon of m_1_ makes the intermediate image of m_1_ and pass it to the recon of m_2_, which uses it for calculating CD image and makes the intermediate image of m_2_ and pass it back to the recon of m_1_. To evaluate the proposed method, phase contrast images were acquired using an axial slice of ascending aorta at the level of the bifurcation of the pulmonary artery. A retrospectively ECG-gated flow-encoded 2D PC MRI pulse sequence was used with typical parameters of: FOV= 320×320 mm^2^, resolution = 2.5ms x 2.5 ms, slice thickness = 6mm, TR/TE=15/6.5ms, flip angle= 30°, temporal resolution = 30ms, VENC = 300 cm/s. The under-sampling was performed retrospectively from a fully sampled data using a Gaussian random under-sampling for rates of 3 and 5. In an IRB approved study, 14 healthy adult subjects (5 males, 17-70 years) were recruited. Reconstructed images were compared with fully-sampled acquisition and CS without CD sparsity (i.e. *λ_CD_*= 0).

## Results

**Figure 2:**
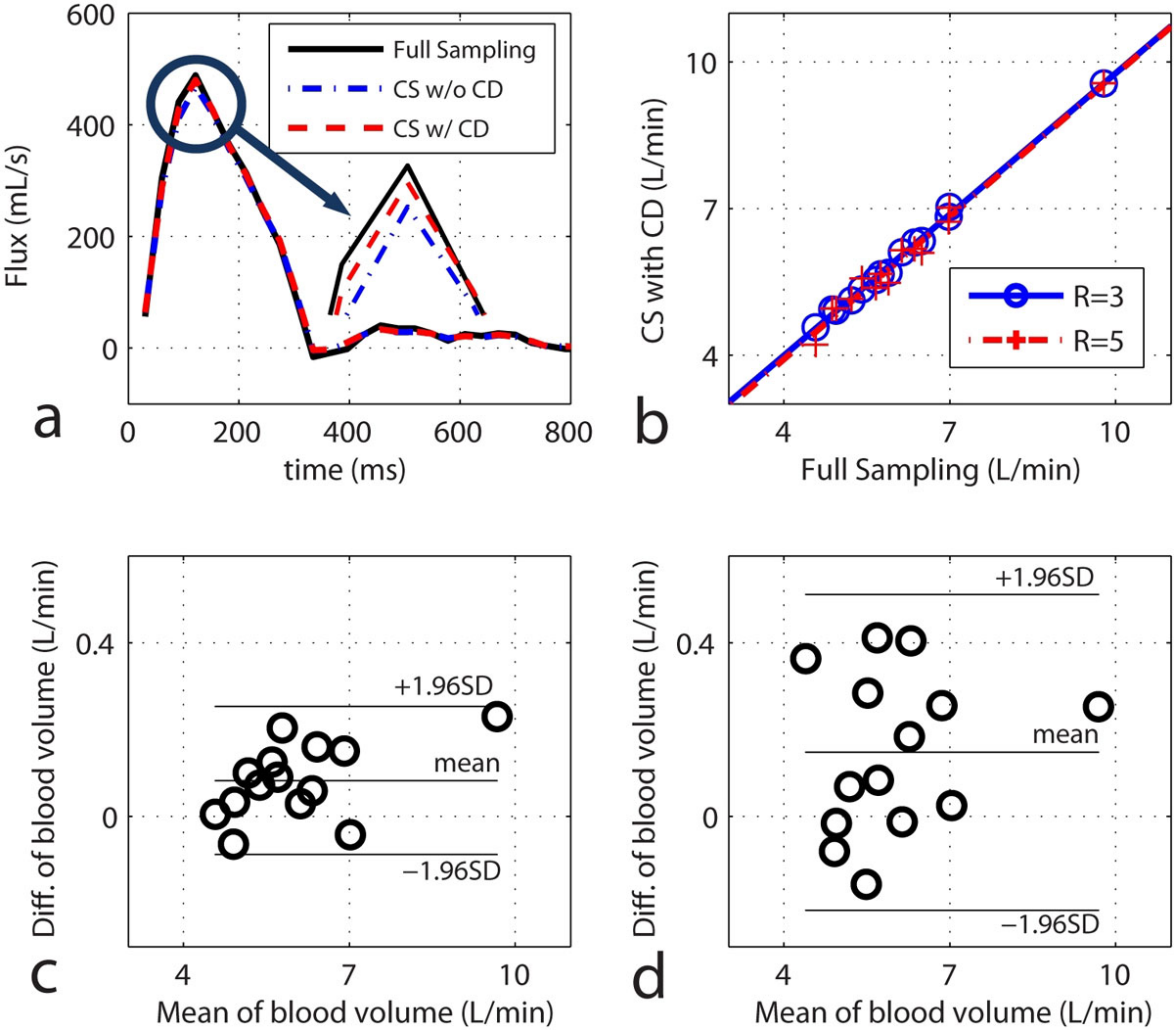
**a**) Example flow rate curves through ascending aorta for acceleration rate of 5, comparing the CS reconstruction method with and without CD sparsity. **b**) linear regression line between the CS reconstruction with CD sparsity and the reconstruction with full samples, **c,d**) Bland-Altman plots for rate 3 and 5 vs. fully sampled acquisition.

Figure 2a shows an example blood volume through the cardiac cycle for fully-sampled and under-sampled acquisition. Figure 2b shows the linear regression relationship of blood volume between fully-sampled vs. under-sampled reconstruction for acceleration rate of 3 and 5. It shows that CS reconstruction with CD gives the comparable volume measurements with the one with full-sampling. The corresponding Bland-Altman graphs are shown in Figure 2c and 2d, which shows excellent agreement between the two techniques.

## Conclusions

In a retrospectively under-sampling study, accelerated CS with complex difference sparsity yields accurate flow measurement for acceleration rate of 3 and 5. Further study using a prospective accelerated acquisition in a larger patient cohort is needed to clinically evaluate this technique.

## Funding

NIH R01EB008743-01A2.
